# Bi-containing Electrolyte Enables Robust and Li Ion Conductive Solid Electrolyte Interphase for Advanced Lithium Metal Anodes

**DOI:** 10.3389/fchem.2019.00952

**Published:** 2020-01-22

**Authors:** Yongliang Cui, Sufu Liu, Bo Liu, Donghuang Wang, Yu Zhong, Xuqing Zhang, Xiuli Wang, Xinhui Xia, Changdong Gu, Jiangping Tu

**Affiliations:** State Key Laboratory of Silicon Materials, Key Laboratory of Advanced Materials and Applications for Batteries of Zhejiang Province, School of Materials Science and Engineering, Zhejiang University, Hangzhou, China

**Keywords:** Li metal anode, K_4_BiI_7_ additive, solid electrolyte interphase, ionic conductivity, dendrite suppression

## Abstract

The notorious lithium dendrite growth, causing the safety concern, hinders the practical application of high-capacity Li metal anodes for rechargeable batteries. Here, a robust and highly ionic conductive solid electrolyte interphase (SEI) layer to protect Li metal anode is *in-situ* constructed by introducing trace additive of tetrapotassium heptaiodobismuthate (K_4_BiI_7_) into electrolyte. The K_4_BiI_7_-added electrolyte enables Li metal anode to display a stable cycling for over 600 cycles at 1.0 mA cm^−2^/1.0 mAh cm^−2^ and over 400 cycles at 5.0 mA cm^−2^/5.0 mAh cm^−2^. *In situ* optical microscopy observations also conform the suppression of Li dendrites at high current density. Moreover, the *in-situ* SEI layer modified Li anode exhibits an average Coulombic efficiency of 99.57% and less Li dendrite growth. The Li-S full sells with the modified electrolyte also show improved electrochemical performance. This research provides a cost-efficient method to achieve a highly ionic conductive and stable SEI layer toward advanced Li metal anodes.

## Introduction

It is widely accepted that the existing battery systems remain insufficient for the continuously urging demand in consumer electronics, electric vehicles and grid storage devices (Yang et al., 2011; Goodenough and Park, 2013; Cheng et al., 2017c). As a promising anode material for energy storage systems, Li metal has a high theoretical capacity (3,860 mAh g^−1^) and the lowest negative electrochemical potential (−3.04 V vs. standard H/H^+^) (Guo et al., 2017; Lang et al., 2017). However, the commercial application of rechargeable Li metal batteries has been hindered by the uncontrolled Li dendrite growth and low Coulombic efficiency during Li plating/stripping (Xu et al., 2014). Due to the high electrochemical activity of metallic Li, parasitic reactions occur spontaneously when fresh Li contacts with most organic electrolytes, inducing a solid electrolyte interphase (SEI) layer on the Li surface (Cheng et al., 2016). The ideal SEI layer should have a high ionic conductivity which can suppress the Li dendrite growth according to Sand's time model (Cheng and Zhang, 2015, 2018). Moreover, the SEI layer should be electronically insulating and electrochemically stable which can promise the Li plating under SEI layer and prevent further reactions between Li metal and electrolyte, respectively (Cheng and Zhang, 2015). For these purposes, new insights into the suppression of Li dendrite growth and stabilization of Li metal have been explored with the rising of advanced material chemistry, including liquid electrolyte additives, highly concentrated electrolytes (Suo et al., 2013; Zheng et al., 2018), *ex-situ* coating (Zhang et al., 2015, 2016; Kim et al., 2018; Shen et al., 2019; Sun et al., 2019) and 3D conductive scaffold composite (Liu et al., 2018a,b,c), etc. Among these strategies, developing new electrolyte additives is the most cost-efficient and can substantially enhance the stability of SEI layer compared with developing physical protective layer, which cannot change the breakage repair mechanism. Previous researches have shown that various electrolyte additives are proved to be beneficial for depressing the growth of lithium dendrites. Different kinds of additives have different mechanisms for lithium dendrites suppression. Some of those additives, such as monofluoroethylene carbonate (FEC) (Zhang et al., 2017), cyclic carbonate trans-difluoroethylene carbonate (DFEC) (Su et al., 2019), halogenated salts (LiF, LiCl, LiBr, LiI) (Lu et al., 2014), can react with metal lithium and produce dense inorganic salts in the SEI to suppress lithium dendrite growth. And some functional additives, such as Cs^+^, Rb^+^ (Ding et al., 2013), KNO_3_ (Sahalie et al., 2019), can realize dendrite-free lithium deposition via self-healing electrostatic shield mechanism. Other organic additives, such as poly (sulfur-random-1,3-diisopropenylbenzene) (PSD) et al. can form a flexible and robust hybrid SEI layer to constrain lithium dendrite formation.

Previous works suggest that ether-based electrolytes can be better choices for LMBs compared with carbonate electrolytes, which are commonly used in commercial lithium-ion batteries (Li et al., 2015). In the ether-based electrolytes, which are commonly used in lithium–sulfur and lithium–air battery systems, the SEI layer formed comprises oligomers due to the existence of 1, 3-dioxolane (DOL) as an electrolyte solvent. The oligomer layer on the Li surface can supply a certain extent of flexibility, which can accommodate the volume change of Li anode (Wang et al., 2018). Although various additives including organic and inorganic compounds have been selected to improve the stability of SEI layers, their effectiveness in suppressing dendrite growth is undermined by the rapid consumption during cycling. For ether-based electrolytes, LiNO_3_ is the most commonly used additive for Li-S full cells, which can oxidize Li metal and solvent molecules to produce a passivation layer on the Li surface to protect the anode from further corrosion (Zhang, 2012; Zhang et al., 2018). However, with LiNO_3_ as sole additive, the improvement of Li anode is limited when the cycling current density is beyond a threshold. This kind of SEI layer cannot maintain its uniformity during long-term cycling (Cheng et al., 2017b). Moreover, the Coulombic efficiency and the electrochemical performance of this SEI layer at high current density are unsatisfactory because of its limited ionic conductivity. According to Sand's time model, when Li ions on the anode surface are fully plated, Li dendrites start to grow, which is also induced by low ionic conductivity (Cheng and Zhang, 2018). Thus, this kind of electrolytes still need further improvement for implementation.

Inspired by the research of introducing electrolyte additives containing metal ions that have lower reactivity with sulfur than lithium to reduce crystallinity of the impurity phases and obtain a highly ion conductive SEI layer (Zu et al., 2016), we proposed a facile strategy to modify the electrolyte by trace addition of Bi-containing additive. Finally, tetrapotassium heptaiodobismuthate (K_4_BiI_7_) was selected because of its high solubility in ether based electrolyte and a modified electrolyte composed of 1 M LiTFSI dissolved in DOL/DME (1: 1 in volume) with 1% LiNO_3_ and 0.5 wt% K_4_BiI_7_ (denoted as ME) was designed and tested in LMBs. At the same time, electrolyte without K_4_BiI_7_ additive (denoted as TE) was also prepared as control sample. Compared with the routine SEI layer formed in K_4_BiI_7_-free electrolyte, the modified SEI possesses a polycrystalline structure with lower crystallinity, which has more grain boundaries and provides more channels for Li ion diffusion (Cheng et al., 2018). The modified SEI layer enabled stable cycling for more than 600 cycles with a voltage hysteresis of ~20 mV at 1.0 mA cm^−2^/1.0 mAh cm^−2^ and over 400 cycles at 5.0 mA cm^−2^/5.0 mAh cm^−2^. *In situ* optical microscopy observations also conform the suppression of Li dendrites at high current density. Moreover, the cells with K_4_BiI_7_-added electrolyte displayed a much higher average Coulombic efficiency of 99.57% during Li plating/stripping. The Li-S full sells with the electrolyte also showed improvement performance during cycling due to the Li-ion conductive nature and high stability of the modified SEI layer.

## Result And Discussion

**Figure 1:**
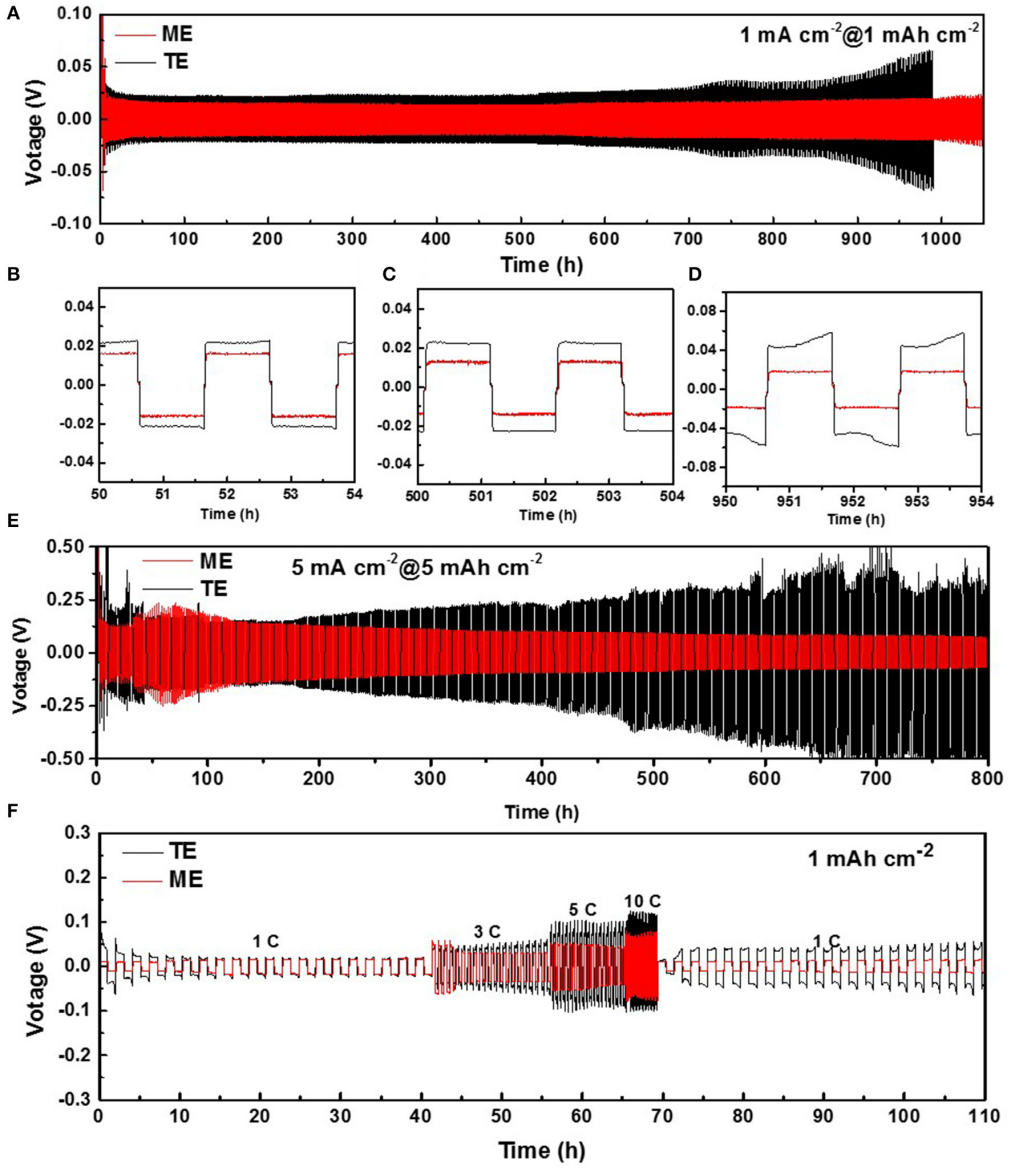
**(A)** Voltage-time curves of Li|Li symmetrical cells at 1.0 mA cm^−2^/1.0 mAh cm^−2^ and **(B–D)** the detailed magnified voltage-time curves at different cycling time. **(E)** Voltage-time curves of Li|Li symmetrical cells at 5.0 mA cm^−2^/5.0 mAh cm^−2^ **(F)** Voltage-time curves of Li|Li symmetrical cells at different current densities.

Firstly, symmetric Li-Li cells are assembled containing the two kinds of electrolytes to investigate the electrochemical stability of Li metal anode. During long-term cycling at plating/stripping current density of 1.0 mA cm^−2^/1.0 mAh cm^−2^, both the routine and modified Li anodes exhibit a stable cycling for 600 h as shown in Figure 1A. This is attributed to the effect of LiNO_3_ which can oxidize Li metal and solvent molecules to form a passivation layer on the surface, and then to protect the anode from further erosion by components of the electrolyte (Wang et al., 2018). For further comparison, the detailed voltage curves at different cycling stages are shown in Figures 1B–D. In the initial and middle cycling stages, the voltage hysteresis of Li-Li cell in K_4_BiI_7_-added electrolyte remains stable at ~18 mV, which is slightly lower than that of normal LiTFSI electrolyte (~22 mV). After 700 h cycling, the modified Li anode still maintains a voltage hysteresis of ~20 mV, whereas for the electrolyte without additive, the voltage hysteresis gradually rises to ~60 mV, which indicates a larger polarization and Impedance increase for SEI. The difference is more obvious when the current density and areal capacity rise to 5.0 mA cm^−2^/5.0 mAh cm^−2^ as shown in Figure 1E. After 800 h (400 cycles), the modified SEI protected Li anode still maintains a voltage hysteresis of 100 mV whereas for the Li anode with a routine SEI, the voltage hysteresis dramatically rises after 200 h. This can be ascribed to the highly resistive layer of routine SEI. Figure 1F shows the voltage hysteresis of symmetric Li-Li cells at different current densities to better reflect the lithium ion migration performance and stability of the SEI layer. When the current density increases to 1, 3, 5, and 10 mA cm^−2^, the voltage hysteresis of Li-Li cell in the K_4_BiI_7_-added electrolyte are 18, 30, 52, and 75 mV, respectively, which is distinctly lower than that in traditional electrolyte (20, 48, 102, and 124 mV). More importantly, when the current density returns to 1 mA cm^−2^, the voltage hysteresis of Li-Li cell in the modified electrolyte remains stable at ~15 mV, which is even lower than that in the initial stage. However, there is an obvious increase in voltage (up to ~42 mV) of Li anodes in routine electrolyte, which indicates a sharp rise of the electrode interphase resistance. The superior Li-ion diffusion behaviors, along with the excellent electrochemical stability, initially confirm that K_4_BiI_7_ can function as an efficient additive for rechargeable Li metal batteries.

**Figure 2:**
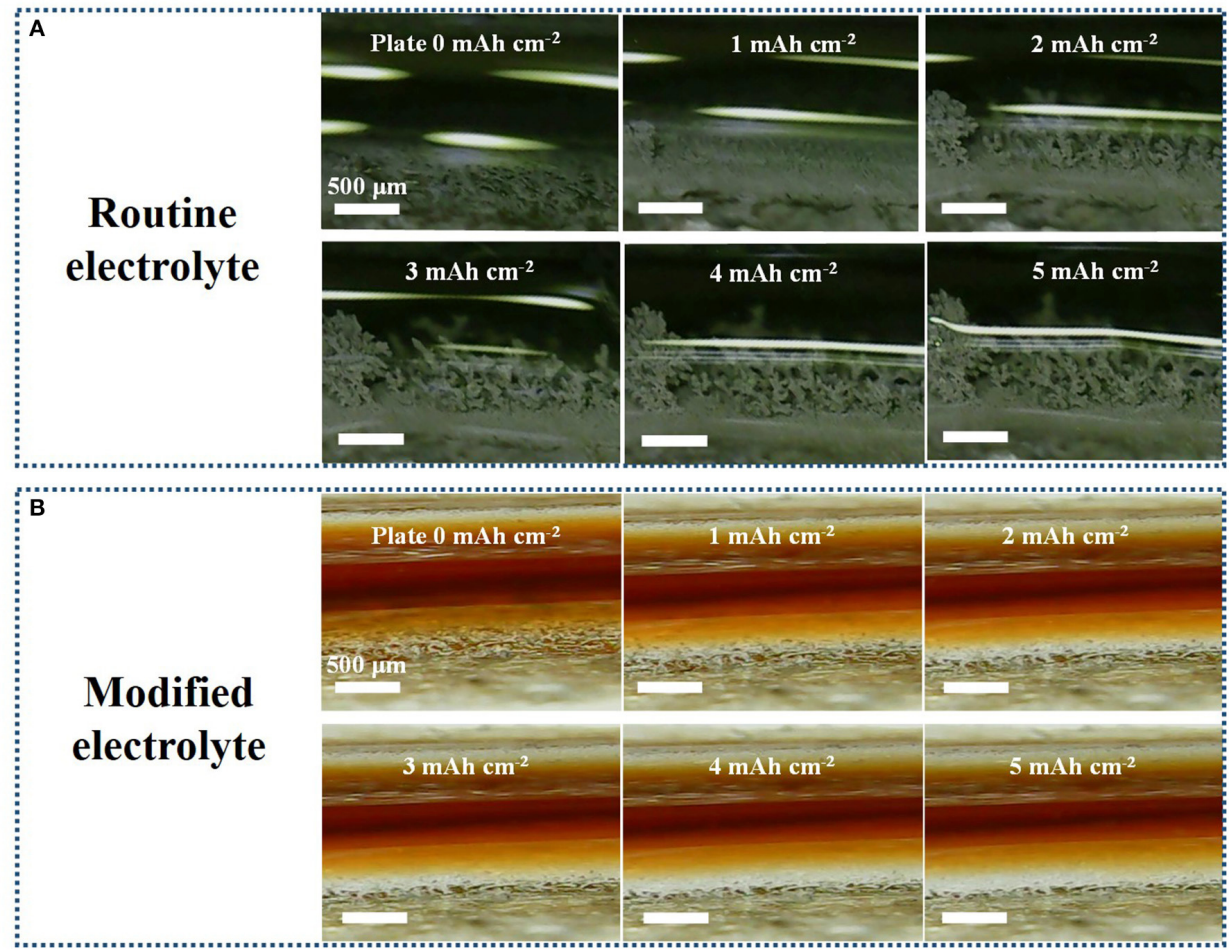
*In situ* optical microscopy observations (captured from the videos) of the electrolyte–electrode interface during electrodeposition in **(A)** routine electrolyte and **(B)** modified electrolyte at an extremely high current density of 60 mA cm^−2^ with 5 min per cycle. The capacity (mAh cm^−2^) of Li being electrodeposited on the electrodes is shown in the top middle of each image.

To check the growth inhibition of Li dendrites, Li electrodeposition was conducted and visualized at all stages by *in situ* optical cell (Figure S2). In our case, a symmetrical cell with bare Li foils as the working and counter electrodes is assembled with a distance of ≈2 mm. Figure 2A shows the morphology evolution of Li electrode interface during electrodeposition at 60 mA cm^−2^ in routine electrolyte. Obviously, the dendritic Li forms after 2 mAh cm^−2^ plated on the Li-metal surface. After the capacity increases to 5 mAh cm^−2^, the growth of Li dendrite becomes much more serious. Despite 5 mAh cm^−2^ Li metal has been subsequently stripped as shown in Figure S1, the dendritic Li still remains almost the same, which would further aggravates Li dendrite during subsequent cycling. As a comparison, morphology of Li electrode in modified electrolyte do not change much and the growth rate of dendrite is greatly suppressed. Figure 2B exhibits Li electrode keeps a smooth surface without any dendrites in the whole electrodeposition stage. During the following Li stripping process, the surface of Li electrode in K_4_BiI_7_-added electrolyte is also much denser than that in routine electrolyte (Figure S1). The microscopic analysis of Li metal anodes is also conducted by comparing scanning electron microscope (SEM) images. The same amount of Li metal is electrochemically deposited onto bare Cu substrates at 1 mA cm^−2^ and their corresponding morphologies are clearly shown in Figure S3. The surface morphology of the Li anode in K_4_BiI_7_-added electrolyte obviously exhibits a columnar and compact structure. And there is no dendrite or crack forming, which reveals the superiority for advanced and safe Li metal batteries. In contrast, the Li plated in traditional electrolyte displays a loose structure of “noodle-like” lithium dendrites, with large volume expansion. When magnified, the surface of Li deposited in the modified electrolyte also presents a flat and homogeneous plane. However, Li nucleus appear on the surface of “noodle-like” lithium dendrites, which reveals a trend to grow more harmful dendrites. These results vividly verify that K_4_BiI_7_-added electrolyte is able to facilitate uniform Li electrodeposition and can effectively prevent the formation of Li dendrites.

The most common method to measure the Li Coulombic efficiency (CE) in a certain electrolyte is to use a Li|Cu cell, which is calculated by the ratio of Li stripped from Cu substrate to that deposited during the same cycle (Qian et al., 2015; Ma et al., 2017). However, the Li loss associated with side reactions and alloying reaction between Li and Cu substrate surface affects the CE of each cycle (Adams et al., 2018; Lin et al., 2019). Herein, we adept a new strategy to eliminate the uncertainty related to substrate surface by calculating average CE (ACE) of Li electrode over *n* cycles (Adams et al., 2018). Figure S4 shows the schematic diagram of cycling Li|Cu cells for measurement of ACE. Firstly, a stabilization process was introduced to stabilize Cu surface by depositing 3 mAh cm^−2^ Li on Cu substrate and striping exhaustively to the cut off voltage (1 V). Then a given amount of charge (*Q*_*T*_ = 3 mAh cm^−2^) was used to deposit Li onto Cu substrate at 0.5 mA cm^−2^ as Li source. After that, a small proportion of this charge (*Q*_*C*_ = 0.5 mAh cm^−2^) was cycled between Li and Cu electrode for *n* (*n* = 100) cycles. Finally, the remaining Li reservoir was stripped exhaustively to the cut-off voltage. The final striping charge (*Q*_*S*_) was measured and the ACE could be calculated by following equation:

$$\begin{array}{l} ACE=\frac{\text{n}{\text{Q}}_{C}+{\text{Q}}_{S}}{\text{n}{\text{Q}}_{C}+{\text{Q}}_{T}} \end{array}$$

**Figure 3:**
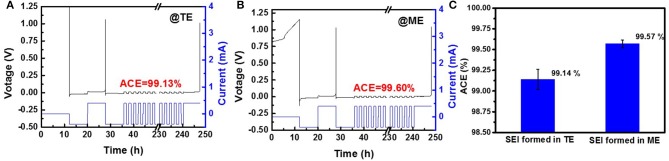
Typical constant current protocol and measured voltage vs. time plot of Li|Cu cells with **(A)** TE and **(B)** ME. **(C)** Average Coulombic efficiency of Li|Cu cells with different electrolytes.

Figures 3A,B show typical constant current protocol and measured voltage vs. time plot of Li|Cu cells with routine electrolyte and modified electrolyte. To make the results more convincing, the results are statically treated and shown in Figure 3C. After 0.5 wt% K_4_BiI_7_ is added into the electrolyte, a relatively high ACE of 99.57% is obtained, which is much higher than that without additive (99.14%). The calculated ACE are the statistical result of five cells tested under the same conditions (Table S1) and the typical results are shown in Figure S2. Such a great difference is a critical evidence to demonstrate that adding K_4_BiI_7_ in the electrolyte can produce a robust SEI which can effectively reduce the side reactions between lithium and electrolyte and thus suppress the growth of Li dendrite.

**Figure 4:**
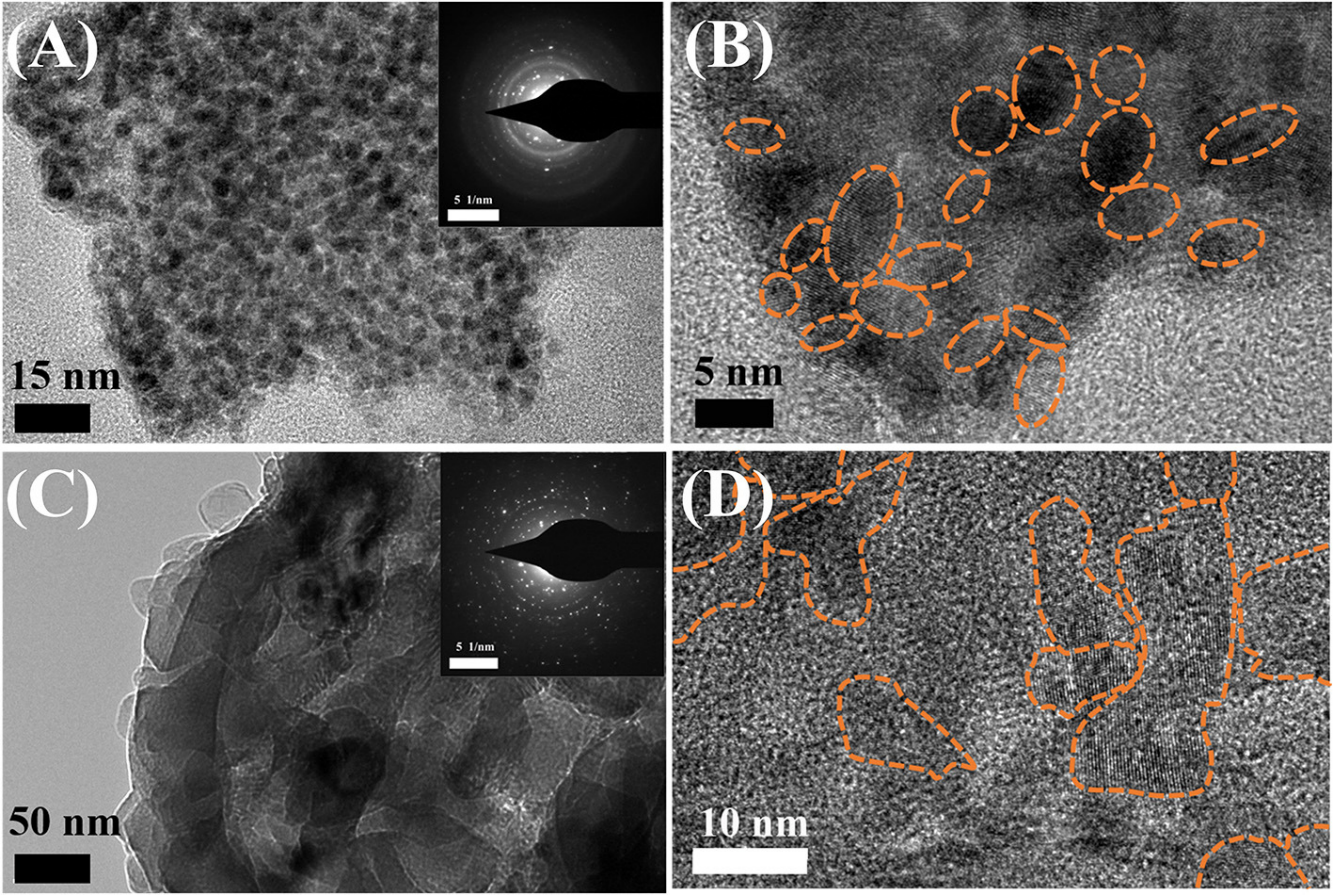
TEM images of the **(A)** routine SEI and **(C)** modified SEI with SAED patterns inserted. High resolution TEM images of the **(B)** routine SEI and **(D)** modified SEI (crystal particles are marked out by dotted circle).

To identify the mechanism behind the outstanding electrochemical performance of Li metal electrode in the K_4_BiI_7_-added electrolyte, high resolution transmission electron microscopy (HRTEM) and selected area electron diffraction (SAED) were carried out to probe the structure of the modified SEI. As shown in Figure 4, both the routine and modified SEI layers show a classic mosaic-like morphology and the SAED patterns validate the polycrystalline nature of the SEI layers (Cheng et al., 2018). There are two notable differences in the morphologies obtained between the modified and routine SEI. First, the grain sizes in the modified SEI (~5 nm) (Figure 4B) is much smaller than that in routine SEI (10 ~ 20 nm) (Figure 4D) as shown in high-resolution TEM images. The SAED images also verify the size difference of the nanoparticles, indicating that the K_4_BiI_7_ additive helps to reduce the crystal size in the modified SEI layer. Secondly, the organic component in routine SEI takes a larger part compared with the modified SEI. And those organic ingredients such as RCOOLi ROCO_2_Li and ROLi (Wan et al., 2018) always exist in amorphous structure. It is believed that the organic components in the SEI layer are not good for the high ionic conductivity (Wood et al., 2016; Liu et al., 2019). Therefore, the additive-modified SEI layer is expected to possess a higher ionic conductivity.

**Figure 5:**
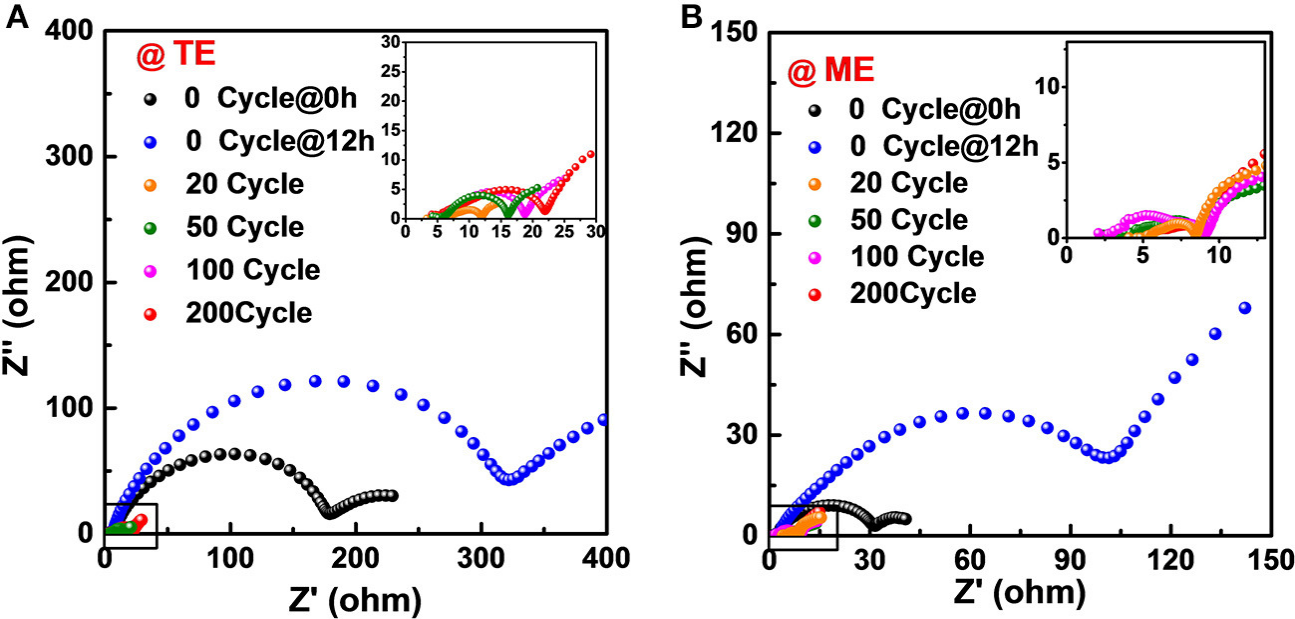
EIS of Li|Li cells with **(A)** TE and **(B)** ME at different cycling stages.

For LMBs with liquid electrolytes, Li ion transfers much faster in electrolyte than that in the solid SEI layer. Therefore, the SEI layer decides Li ion transfer behavior of the whole cell. Figure S5 shows that the conductivity difference of the routine electrolyte and the K_4_BiI_7_-added electrolyte is negligible. To confirm the high conductivity of the modified SEI, electrochemical impedance spectroscopy (EIS) measurement was conducted. Figure 5 shows the Nyquist plots of Li symmetrical cells containing different electrolytes at different charge and discharge stages. All the Nyquist plots exhibit a semicircle in the high frequency region, which is well recognized to reflect Li ion migration through the SEI on Li surface (Wan et al., 2018). Before cycling, the interfacial resistances of the cells increase over time, indicting continuous side reactions between Li metal and electrolyte. However, the Li Li symmetrical cell with ME shows a much lower interfacial resistance of 30 Ω after 0 h rest and 105 Ω after 12 h rest than that with TE (175 and 320 Ω, respectively). This can be attributed to the stable electrode interphase established by the efficient reaction between Li and Bi-containing additive. And the dramatically reducing interfacial impedance drops dramatically upon cycling because the formation of a suitable SEI layer occurs (Markevich et al., 2017). The Li symmetrical cell shows an interfacial resistance of ~8 Ω in K_4_BiI_7_-added electrolyte while the other one has an interfacial resistance of 12 Ω in K_4_BiI_7_-free electrolyte after 20 cycles, indicating that the modified SEI layer is more conductive for Li ions (Lu et al., 2019). More importantly, after cycling for 50, 100, 200 cycles, the electrochemical impedance of Li anode in electrolyte without additive shows an expanded semicircle at high frequency because of the continuous Li dendrite growth, whereas in K_4_BiI_7_-free electrolyte maintains its stable value as low as 9 Ω, indicating a more homogenous and electrochemically stable SEI layer is successfully built by the *in situ* reaction between Li metal and K_4_BiI_7_ additive.

**Figure 6:**
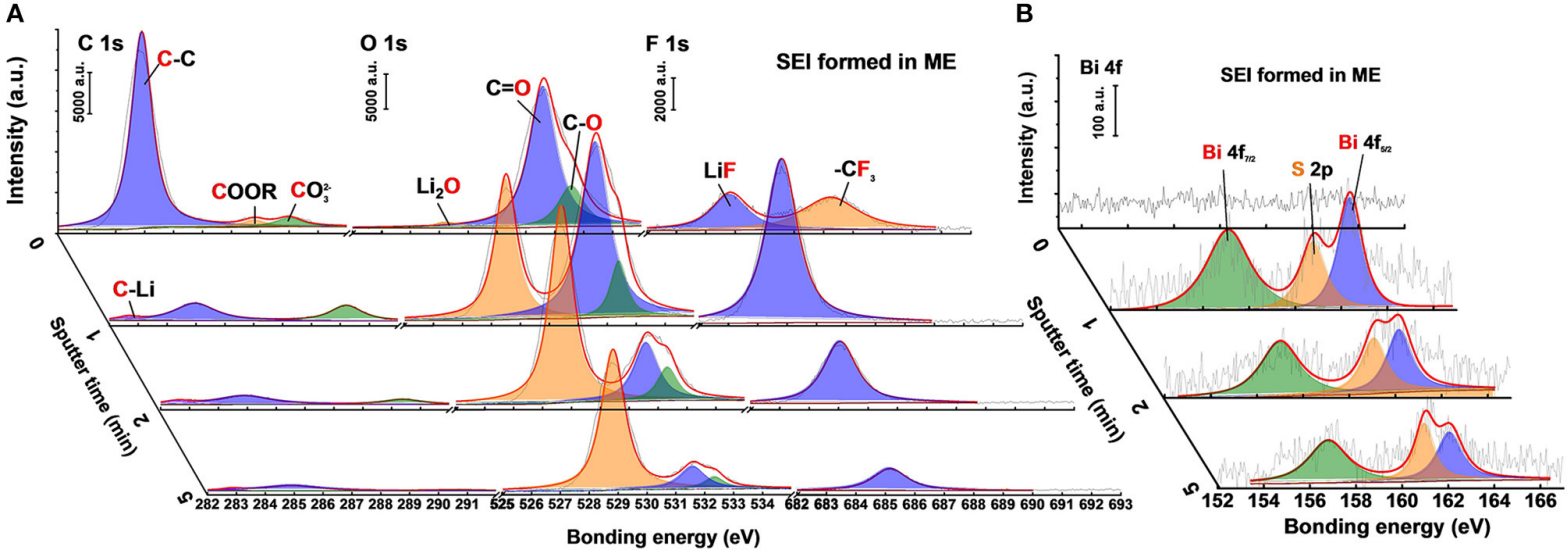
XPS spectra of **(A)** C 1s, O 1s, F 1s and **(B)** Bi 4f species at various depths of the modified SEI on Li anodes after 20 cycles in Li|Li batteries.

X-ray photoelectron spectroscopy (XPS) was employed to analyze the specific compositions of the SEI layer after 20 cycles in the Li|Li batteries. Figure 6 shows the composition change of SEI layer formed in the K_4_BiI_7_-added electrolyte by using XPS depth profiles. After 20 cycles, the C 1s spectrum of the SEI layer shows typical decomposition products of solvents including ${\text{CO}}_{3}^{2-}$, COOR and C-C corresponding to previous reports (Cheng et al., 2017a; Kozen et al., 2017). However, their fractions dramatically decrease after Ar ion sputtering, indicating that the organic components mainly exist on the upper surface. This result can also be confirmed by the O 1s and F 1s spectra (Figure 6A). The main compositions on the upper layer of SEI are C = O, C-O, and –CF_3_, which are related to the decomposition of DOL and LiTFSI salt (Li et al., 2017a,b). Inorganic ingredients such as Li_2_O, LiF, and LiCO_3_ compose most of the composition below the surface (Figure 6A). Overall, the SEI layer formed in the normal 1 M LiFSI electrolyte has similar composition (Figure S6), including inorganics and organics. The main difference exists in Bi 4f spectra is shown in Figure 6B. The Bi 4f_7/2_ (157.1 eV) and Bi 4f_5/2_ (162.4 eV) peaks which belong to Bi metal are obviously detected below the organic surface of SEI formed in ME. Part of the S 2p peak is also detected because of the partial overlap of their bonding energy range. Thus, in the optimized electrolyte system, the SEI layer on Li metal surface is modified by the Bi-containing additive. The Bi metal in the SEI layer comes from the substitution reaction between Bi^3+^ and Li metal as shown in Equation (1). Because of the lowest negative electrochemical potential of Li metal, Li-Bi alloy will form after a period of contact displayed in Equation (2), which can also serve as a fast Li ion transmission medium (Liang et al., 2017).

(1) $$\begin{array}{l} {\text{Bi}}^{3+}+3\text{Li}\to \text{Bi}+3\text{L}{\text{i}}^{+} \end{array}$$

(2) $$\begin{array}{l} xLi+yBi\to {\text{Li}}_{\text{x}}{\text{Bi}}_{\text{y}} \end{array}$$

It can be concluded that the K_4_Bi_3_I_7_ additive in the electrolyte can significantly increase the ionic conductivity of SEI layer (Figure S7). After fast reaction with Li metal, Bi^3+^ is reduced into Bi metal particles, which act as “nanopins” to interrupt the growth of other inorganic crystals (Zu et al., 2016). This is attributed to the rapid formation of Bi metal inserted in the Li anode, which can regulate the crystal growth during SEI formation. Therefore, the grain size of individual components is reduced and a mosaic SEI layer with poor crystallinity is formed. It is believed that enriched grain boundaries can afford more channels for Li-ion diffusion, leading to an increase in ionic conductivity (Zu et al., 2016; Cheng et al., 2018).

**Figure 7:**
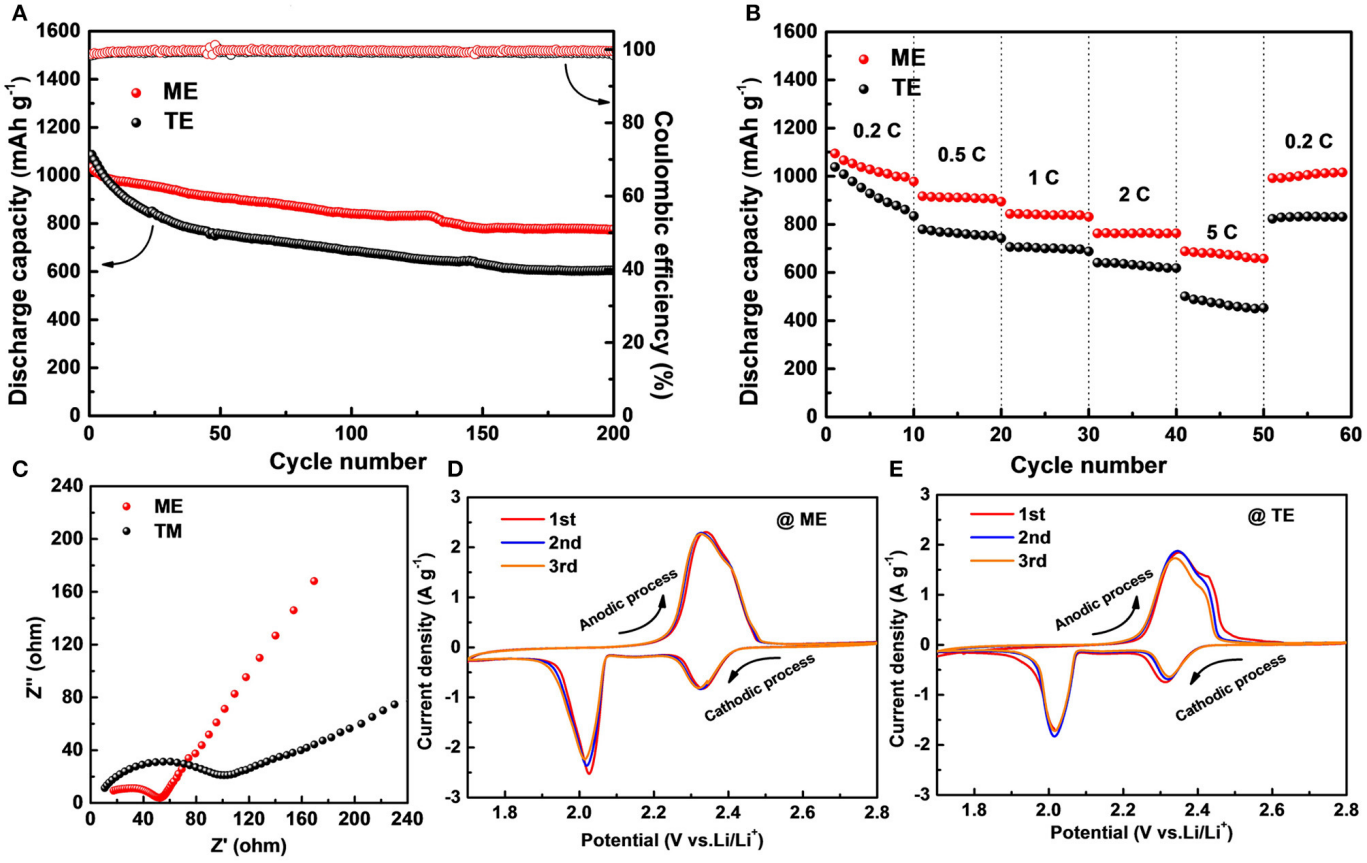
Electrochemical performance of Li|S full cells with different electrolyte. **(A)** Cycling performance of at 0.2C. **(B)** Rate capability. **(C)** Nyquist plots before cycling. CV curves for the initial three cycles of cells with **(D)** ME and **(E)** TE at a scan rate of 0.1 mV s^−1^.

Moreover, to investigate the practical application of the modified electrolyte, Li-S full cells are assembled. As shown in Figure 7A, the cell containing traditional electrolyte delivers an initial capacity of 1,085 mAh g^−1^ but only retains 605 mAh g^−1^ after 200 cycles at 0.2 C. Although the initial capacity of cell with K_4_BiI_7_-added electrolyte presents a similar value of 1,034 mAh g^−1^, the remained capacity after 200 cycles is about 776 mAh g^−1^. The capacity retention rate of cell with modified electrolyte is 75.05% corresponding to a low degradation rate of 0.125% per cycle, which is superior to that of cell containing traditional electrolyte (57.6, 0.221%). Moreover, the K_4_BiI_7_-added electrolyte produces a high Coulombic efficiency of 99.65%, higher than the K_4_BiI_7_-free electrolyte (99.18%). This is mainly attributed to the robust SEI layer formed on Li anode that prevents side reactions between Li metal and polysulfides and thus suppresses the serious shuttle effects (Zhao et al., 2019). Figure 7B shows the rate capacity which reflects the charge and discharge performance at different current densities. The cells with K_4_BiI_7_-added electrolyte achieve average discharge capacities of 1,028, 910, 840, 763, and 674 mAh g^−1^ upon cycling at programed current densities of 0.2, 0.5, 1, 2, 5 C, respectively. Impressively, when the current returns to 0.2 C, the capacity rises back to 1,004 mAh g^−1^, nearly 97.6% of the capacity in the initial stage. In contrast, the cells exhibit inferior rate performance without K_4_BiI_7_ additive (only 89.4% of the initial capacity when the current density falls back to 0.2 C). The improvement of rate performance can be attributed to the enhanced ionic conductivity of the modified SEI layer. In order to verify this point of view, EIS analysis of Li|S cells were conducted as shown in Figure 7C. According to the Nyquist plots, the cell containing K_4_BiI_7_-added electrolyte displays low charge-transfer resistance and Warburg impedance. The smaller semicircle at the high-frequency region exhibits the improvement of the charge transfer (Zhong et al., 2018). At the low frequency region, the more vertical straight line presented in the K_4_BiI_7_-added cell reveals the faster ion diffusion paths (Ganesan et al., 2016; Wang et al., 2017). The K_4_BiI_7_ additive in the electrode can increase both ion transfer in SEI on the anode and charge transfer ability of the cathode. Figures 7D,E shows the typical CV curves of the C/S electrode in a potential range of 1.7–2.8 V (Li/Li^+^) at a scan rate of 0.1 mV s^−1^. Both kinds of the Li|S cells reveal two characteristic reduction peaks in the cathodic scan and one oxidation in the anodic scan (Guo et al., 2018a,b). Compared with the cell without K_4_BiI_7_ additive, the electrolyte-modified cell shows high peak intensities and large enclosed area, indicating high sulfur utilization and fast reaction kinetics. Moreover, the highly overlapped curves during three scan cycles demonstrate that the electrolyte with additive can stabilize the charge and discharge performance. The above results indicate that the electrolyte modification by adding K_4_BiI_7_ additive can stabilize Li metal interface and suppress Li dendrite growth, which also contributes to the improved Coulombic efficiency in Li-S full cells.

## Conclusion

In summary, we propose a new Bi-containing additive in ether electrolyte for high stable and efficient dendrite-free Li metal anodes by forming a highly ionic conductive SEI layer. The additive can help to yield a polycrystalline mosaic-like SEI layer with poor crystallinity and rich grain boundaries. This structure renders an enhanced Li ion conductivity to suppress Li dendrite growth and dramatically improve the average Coulombic efficiency of Li metal anodes. The modified SEI layer enables stable Li plating/stripping for over 600 cycles at 1.0 mA cm^−2^/1.0 mAh cm^−2^ with a voltage hysteresis of ~20 mV and over 400 cycles at 5.0 mA cm^−2^/5.0 mAh cm^−2^. This SEI layer can also endure an extremely high current density of 10 mA cm^−2^ for cycling. *In situ* optical microscopy observations also conform the suppression of Li dendrites at high current density. After pairing with sulfur cathode in full cells, the modified electrolyte exhibits enhanced performance. Therefore, our research provides a cost-efficient method to achieve a highly Li-ion-conductive SEI layer toward advanced Li metal anodes, especially for the next-generation Li-S full batteries.

## Experimental Section

### Materials Preparation

The ether based electrolyte was prepared by dissolving 1 M Lithium bis(trifluoromethanesulfonyl)imide (LiTFSI, 99.95%, Sigma Aldrish) and 1 wt% LiNO_3_ (99.99%, Aladdin) in anhydrous DOL and DME (1:1 by volume, Aladdin). The pre-weighted K_4_BiI_7_ (>99%, Aladdin) additive was added to the prepared electrolyte, followed by magnetic stirring for 3 h to form a stable clarified solution. As a contrast, the electrolyte without additive was also prepared as comparison.

The C/S composite was prepared by a melt-diffusion method: carbon powder (Cabot 2000) and sublimed sulfur (Yongjia, CP) were grinded and mixed in a mortar with a weight ratio of 3:7. The mixture was sealed in an autoclave with a Teflon liner and maintained at 155°C for 12 h. To obtain the sulfur cathode, the C/S composite was mixed with carbon black and polyvinylidene fluoride (PVDF) binder with a weight ratio of 8:1:1 in N-methyl-2-pyrrolidone solvent to yield a slurry, which was coated onto an aluminum foil and dried in a vacuum oven at 60°C for 24 h.

### Materials Characterization

The morphologies of Li anode were observed by a field emission scanning electron microscope (SEM, Hitachi SU8010). Field emission transmission electron microscope (TEM, JEOL JEM-2100F) was used to characterize the crystal structures and particle sizes of SEI layer. The Li metal surface was performed with a X-ray photoelectron spectrometer (XPS, ESCALAB 250Xi, Al Kα X-ray) and all the Li samples were sputtered by 2,000 eV Ar^+^ for 0, 60, 120, and 300 s to probe composition change in the depth of SEI layer. A home-designed container filled with Ar was prepared to transfer all samples containing Li metal to avoid oxidation and parasitical reactions.

### Electrochemical Measurements

CR2025 type coin cells were assembled for electrochemical tests in an argon-filled glove box. Lithium metal foil (500 μm, China Energy Lithium Co., Ltd) was used as anodes and cut into round discs with a diameter of 15 mm. Polypropylene microporous film (Cellgard 2325) served as separator. The electrochemical performances were measured on a LAND battery program-control test system. Li-Cu cells (using 15 mm copper disc as cathodes) and Li symmetrical cells, both loaded 50 μL electrolyte, were assembled for average Coulombic efficiency (ACE) measurement and galvanostatic discharge-charge test separately. The sulfur mass loaded on each sulfur cathode was about 1.5 mg cm^−2^, and the electrolyte-to-sulfur (E/S) ratio is 20 μL mg^−1^. CHI 660D electrochemical workstation was used for cyclic voltammetry (CV) measurements at a scan rate of 0.1 mV s^−1^. *In situ* optical observation is carried out by a quartz cell with transparent quartz window (Kejing, STC-Q), which is assembled in an argon-filled glovebox. Optical microscope camera (Belona, 200X-800X) with Wi-Fi box was applied to visualize Li deposition process. The applied current was set at 60 mAcm^−2^ with electrochemical deposition or dissolution time of 300 s. Electrochemical impedance spectroscopy (EIS) tests were conducted on a PARSTAT MC electrochemical workstation in the frequency range from 10 kHz to 10 mHz at an AC signal of 5 mV.

## Data Availability Statement

All datasets generated for this study are included in the article/Supplementary Material.

## Author Contributions

YC: research concept and design experiment, conduct of experiment, collection of data, data analysis and interpretation, writing the Result and Discussion section of the article. SL: research concept and design experiment, critical revision of this article, data analysis and interpretation. BL, XW, XX, and CG: research concept and design experiment, critical revision of this article. DW: research concept and design experiment, writing the Introduction section of the article. YZ and XZ: research concept and design experiment, data analysis and interpretation. JT: research concept and design experiment, critical revision of this article, final approval of article.

### Conflict of Interest

The authors declare that the research was conducted in the absence of any commercial or financial relationships that could be construed as a potential conflict of interest.
