# Supplementary material for: Bi-containing Electrolyte Enables Robust and Li Ion Conductive Solid Electrolyte Interphase for Advanced Lithium Metal Anodes
Source: Front Chem. 2020 Jan 22;7:952. doi: 10.3389/fchem.2019.00952 (PMC6990125; doi:10.3389/fchem.2019.00952)
Supplement: Supplementary file 1 [file Data_Sheet_1.docx]

Supplementary Material

Bi-containing additive enables robust and Li ion conductive solid electrolyte interfaces on dendrite-free lithium metal anodes

Yongliang Cui, Sufu Liu, Bo Liu, Donghuang Wang, Yu Zhong, Xuqing Zhang, Xiuli Wang, Xinhui Xia, Changdong Gu, Jiangping Tu*

State Key Laboratory of Silicon Materials, Key Laboratory of Advanced Materials and Applications for Batteries of Zhejiang Province, School of Materials Science and Engineering, Zhejiang University, Hangzhou 310027, China

**Figure S1.** In situ optical microscopy observations (captured from the videos) of the electrolyte–electrode interface during striping in (A) routine electrolyte and (B) modified electrolyte at an extremely high current density of 60 mA cm^−2^ . The capacity (mAh cm^−2^) of Li being electrodeposited on the electrodes is shown in the top middle of each image.

**Figure S2.** Visualization setup for Li metal electrodeposition by in-situ microscope.

**Figure S3.** SEM images of Li deposits in (A) (C) ME and (B) (D) TE after continuous plating for 3 h at 1.0 mAh cm^−2^ in Li | Cu cells.

**Figure S4.** Schematic diagram of cycling Li | Cu cells for accurate measurement of Li average Coulombic efficiency.

**Figure S5.** XPS spectra of C 1s, O 1s and F 1s species at various depths of the routine SEI on Li anodes after 20 cycles in Li | Li batteries.

**Figure S5.** The impedance spectra obtained to determine the ionic conductivity of the (A) TE and (B) ME. (C) The calculated conductivity of TM and ME at different temperatures.

The ionic conductivity σ was calculated based on the following equation:

$$\sigma=\frac{l}{R_{b}\cdot S}$$

Where *l* represents the distance between the electrodes, *R_b_* represents the bulk resistance obtained by EIS measurement, and *S* is the cross-sectional area of the blocking electrode. Coin cells with two copper discs (d = 10mm) as the blocking electrodes and a 25 μm-thick separator (Cellgard 2325, Porosity = 39%) are assembled for the impedance test at different temperatures. Then the conductivity is calculated from the spectra.

**Figure S6.** XPS spectra of C 1s, O 1s and F 1s species at various depths of the routine SEI on Li anodes after 20 cycles in Li | Li batteries.

**Figure S7.** Schematic diagrams describing the structures and effects of modified SEI and routine SEI on working Li anode.

**Table S1.** ACE results of 5 cells containing different electrolytes tested under the same conditions.

| ACE/% | Cell 1 | Cell 2 | Cell 3 | Cell 4 | Cell 5 |
| --- | --- | --- | --- | --- | --- |
| Cells with TE | 99.00 | 99.13 | 99.10 | 99.33 | 99.14 |
| Cells with ME | 99.49 | 99.59 | 99.60 | 99.57 | 99.60 |
